# Essential Oil of *Foeniculum vulgare* Mill. as a Green Fungicide and Defense-Inducing Agent against Fusarium Root Rot Disease in *Vicia faba* L.

**DOI:** 10.3390/biology10080696

**Published:** 2021-07-22

**Authors:** Mona M. Khaleil, Maryam M. Alnoman, Elsayed S. Abd Elrazik, Hayat Zagloul, Ahmed Mohamed Aly Khalil

**Affiliations:** 1 Botany and Microbiology Department, Faculty of Science, Zagazig University, Zagazig 44519, Egypt; 2 Biology Department, Faculty of Science, Taibah University, Al-Sharm, Yanbu El-Bahr 46429, Saudi Arabia; mnaaman@taibahu.edu.sa; 3 Plant Protection and Biomolecular Diagnosis Department, Arid Lands Cultivation Research Institute, City for Scientific Research and Technology Applications New Borg EL-Arab, Alexandria 21934, Egypt; elsayed22003@yahoo.com; 4 Chemistry Department, Faculty of Science, Taibah University, Yanbu El Bahr 46429, Saudi Arabia; hdzaghloul@taibahu.edu.sa; 5 Botany and Microbiology Department, Faculty of Science, Al-Azhar University, Cairo 13759, Egypt

**Keywords:** *Foeniculum vulgare*, chitinase, defensin, qRT-PCR, *Vicia faba*, plant disease, root rot, essential oil, plant promotion

## Abstract

**Simple Summary:**

Plant extracts, including essential oils, are a viable alternative method for controlling plant diseases. This work deals with the exploitation of fennel seed essential oil (FSEO) to inhibit *Fusarium solani* and control *Fusarium* root rot disease in *Vicai faba*. In vitro FSEO inhibited mycelium growth by up to 80% at 400 µL/mL of FSEO. In vivo, the protective effects against *Fusarium* root rot disease were recorded when FSEO was applied to *Vicia faba* seeds. The FSEO reduced the disease severity from 98% in plants grown in infested soil with *Fusarium solani* to 60.1% in plants that previously had their seeds treated with FSEO. GC-MS spectrometry analyses showed that the major chemical components in the essential oil were D-limonene, menthol, estragole and 2-decenal. Applications of the essential oil resulted in increased total phenolic and flavonoid contents in leaves compared with untreated inoculated (control) plants. The defense-related genes, such as defensin and chitinase, were differentially expressed. This study revealed that the essential oil of fennel seed was effective as a control agent against *Fusarium* root rot in broad beans.

**Abstract:**

*Fusarium solani*, the causative agent of root rot disease is one of the major constraints of faba bean (*Vicia faba* L.) yield worldwide. Essential oils have become excellent plant growth stimulators besides their antifungal properties. *Foeniculum vulgare* Mill. (fennel) is a familiar medicinal plant that has inhibitory effects against phytopathogenic fungi. Herein, different concentrations of fennel seed essential oil (FSEO) (12.5, 25, 50, 100, 200 and 400 μL/mL) were examined against *F. solani* KHA10 (accession number MW444555) isolated from rotted roots of faba bean in vitro and in vivo. The chemical composition of FSEO, through gas chromatography/mass spectroscopy, revealed 10 major compounds. In vitro, FSEO inhibited *F. solani* with a minimum inhibitory concentration (MIC) of 25 µL/mL. In vivo, FSEO suppressed *Fusarium* root rot disease in *Vicia faba* L. by decreasing the disease severity (61.2%) and disease incidence (50%), and acted as protective agent (32.5%) of *Vicia faba* L. Improvements in morphological and biochemical parameters were recorded in FSEO-treated faba seeds. Moreover, the expression level of the defense-related genes defensin and chitinase was noticeably enhanced in treated plants. This study suggested using FSEO as a promising antifungal agent against *F. solani* not only to control root rot disease but also to enhance plant growth and activate plant defense.

## 1. Introduction

*Vicia faba* L., commonly known as faba bean or broad bean, is an economical legume grain that widely contributes to human consumption, animal fodder and silage making [1]. Faba beans have high nutritional value due to their high protein content, minerals, vitamins and considerable amounts of bioactive compounds [2]. This crop is usually planted at the end of summer. *Vicia faba* is susceptible to several diseases that reduce their yield, especially in moist conditions. Faba beans are attacked by a common disease known as root rot, which is caused by many fungal species including *Fusarium solani*, *Rhizoctonia solani* and *Sclorotium rolfsi* [2,3,4]. *Fusarium solani* is one of the most pathogenic fungi that deteriorate the quality and quantity of many crops’ production [5]. *F. solani* is a common plant pathogen that invades a wide range of hosts including 111 plant species belonging to 87 genera [6]. Globally, *Vicia faba* L. is one of the most common plants suffering from root rot disease. *Fusarium* root rot takes place at the beginning of the growing season, resulting in the death of seedlings [4]. Otsyula et al. [7] reported that, the yield of common beans decreased by up to 84% due to root rot induced by *F. solani*. Management of *Fusarium* root rot is a complex task due to the soil-borne pathogens being located near the rhizosphere and their long-term survival by producing resistant spores [8]. Although there are fungicides that can control fungal diseases, they have negative impacts on human health, the ecosystem and evolving fungicide-resistant strains [9]. Therefore, the use of natural alternatives is the only reliable source for controlling plant diseases [10]. Essential oils (EOs) are among the most important alternatives that play a vital role in plant protection and food preservation, with a wide variety of applications. Several studies have confirmed the use of EOs to manage plant pathogenic fungi and improve the safety and quality of crops [11,12]. The chemical composition of EOs contains many bioactive molecules that have antifungal, antibacterial and antioxidant activity [13]. EOs have many antagonistic effects against bacteria and fungi, as they drive the plasma membrane to lose its ability to act as a barrier, followed by the release of intracellular components and suppression of cellular respiration with homeostasis failure [14]. Moreover, EOs can inhibit the formation of fungal cell walls and electron transport in the mitochondria [15]. Remarkably, one of the important actions of EOs on the plasma membrane is to suppress the secretion of toxins [16]. The usage of EOs effectively enhances the safety and quality of cereals and food products [11]. Regardless of antimicrobial activity, applying essential oils as biocides has many benefits beside antimicrobial activity, including pre-harvest restrictions, being non-toxic for human health, being reliable to use in any type of lands and their convenience for all types of cultivation, such as organic systems [11]. Herbs and aromatic plants are usually used for medicinal purposes and for inhibiting microbes, since they contain essential oils [17]. The fennel herb (*Foeniculum vulgare* Mill.), a member of the *Apiaceae* family, is an important medicinal plant found almost all over the world [18]. It is used as a carminative, antiseptic, diuretic, digestive and expectorant, aiding anticancer, anti-inflammatory, antimicrobial, and antioxidant activities [19]. The leaves and fruits of fennel are used in cosmetics and flavoring substances, while seed extracts exhibit antifungal effects against *Aspergillus* sp., *Candida* sp., *Sclerotinia sclerotiorum* and many other phytopathogens and dermatophytes [18,19]. The main components of fennel oil are trans-anethole (53.51%), carvacrol (11.93%), fenchone (8.32%) and thymol (8.11%) [20]. The essential oil of *F. vulgare* is enriched with phytochemicals such as polyphenols that give it antimicrobial and antioxidant properties [21]. The use of EOs can move from pathogen suppression to plant protection, presumably by different strategies; one such mechanism is defensin, a term used in the description of antimicrobial and antifungal proteins (AFPs), isolated from mammals, insects and plants, and serving as effectors molecules of innate immunity, providing an efficient initial defense against infectious pathogens [22]. Another such strategy is the synthesis of pathogenesis-related (PR) proteins such as chitinases, which hydrolyze chitin, a linear polymer of β-1,4-linked *N*-acetylglucosamine residues that is one of the primary cell wall components of many pathogenic fungi [23,24]. The level of expression of PR genes such as chitinase and defensin increases the defensive response of plants against a wide range of pathogens [25]. Although FSEO has been used to inhibit some fungal species, several studies are still required to discover its efficiency against fungal plant pathogens and plant diseases, and its effect on plant quality and resistance. In addition, there is an increasing demand for this green, natural and safe product for future approaches to crop protection and organic farming. In this context, this study planned to investigate the antifungal potential of FSEO as a fungicide to overcome the economically damaging *Fusarium* root rot disease of *Vicia faba* L. in vitro and in vivo. Furthermore, the effect of FSEO was assessed on plant growth, antioxidant enzymes and the expression levels of defensin genes in *Vicia faba* L.

## 2. Materials and Methods

### 2.1. Plant Material and Extraction of Fennel Seed Essential Oil

*Foeniculum vulgare* (fennel) seeds were obtained from a local market (El-Hawag Company, Cairo, Egypt), and identified and authenticated by the Department of Botany, Faculty of Science, Mansoura University, Egypt. Fifty grams of fennel seeds was air dried and ground into a fine powder and then placed in a 2000 mL flask with 500 mL of distilled water and extracted by the hydro-distillation process using a Clevenger-type apparatus for 5 h to extract the essential oils. The oils were collected in a 250 mL conical flask, dried over anhydrous sodium sulphate and kept at 4 °C until use [26].

### 2.2. Gas Chromatography/Mass Spectral Analysis

The chemical composition of the essential fennel oil was determined by gas chromatography-mass spectrometry system (GC-MS-QP 2010, Shimadzu, Japan), equipped with a flame-ionization detector (FID) with a Rtx-5MS column (30 m × 0.25 mm, 0.25 μm thickness). The essential oil (10 μL) was dissolved in acetone (100 μL) and 1 μL of the solution was injected into the GC/MS system with the following properties: helium was the carrier gas, used at a flow rate of 1 mL/min; split mode (1:25), with 1 μL (1/10 in acetone, *v*/*v*) as the injected volume and 300 °C as the injection temperature. The mass spectra of the obtained compounds were matched with those in the NIST11 library (Gaithersburg, MD, USA) [27].

### 2.3. Isolation of Fusarium solani, Pathogenicity Test and Cultivation

*Fusarium solani* was isolated in the laboratory from infected roots of faba bean plants (*Vicia faba* L.) displaying external signs of rot root disease. The plants were collected from agricultural areas in the production region of Behera, Egypt, in the winter of 2019. *F. solani* isolation was achieved by cutting the infected root into pieces (2 to 3 mm). The fragments were surface-sterilized with a 10% sodium hypochlorite solution for 2 min, then rinsed with sterile distilled water ternary. Pieces were cultured aseptically onto a *Fusarium* selective medium—Nash-Snyder agar (1 g/L KH_2_PO_4_, 0.5 g/L MgSO_4_-7H_2_O, 15 g/L peptone, 20 g/L agar, 1 g/L pentachloronitrobenzene, 0.3 g/L streptomycin sulfate, 0.12 g/L neomycin sulfate) [28]—and incubated at 25 ± 2 °C for 5–7 days. The fungal mycelium was sub-cultured on Czapek-Dox agar medium (CZA) (30 g/L sucrose, 3 g/L NaNO_3_, 0.5 g/L KCl, 100 mg/L FeSO_4_-7H_2_O, 0.5 g/L MgSO_4_-7H_2_O, 1 g/L K_2_HPO_4_). Morphological features as well as microscopic characteristics were investigated [29]. Moreover, molecular identification was also applied; the universal primers ITS1/ITS2 for the ribosomal internal transcribed spacer (ITS) were used. The sequence was compared with the suggested species using the BLAST sequence analysis tool and was registered into GenBank under the accession number MW444555. Koch’s postulate was implemented to confirm that the symptoms of root rot belonged to *F. solani* KHA10 [30]. Eventually, cultures attained from single spores were maintained on CZA and kept at 4 °C for further use. The pure culture has been placed in the culture collection of the Botany and Microbiology Department, AUC (No. BMS0023).

### 2.4. In Vitro Evaluation of Antifungal Activity and Growth Inhibition

#### 2.4.1. Agar Well Diffusion Method

The antifungal activity of FSEO was tested by the well diffusion method with minor modifications. *F. solani* was inoculated on a Czapek-Dox (CZ) broth medium and then incubated at 25 ± 2 °C for 5–7 days [13]. Fungal inoculum of *F. solani* was spread on the surface of CZA plates. Next, 5 wells 8 mm in diameter were made using a sterile cork-borer on each agar plate (90 mm). The wells were filled with 100 µL of different concentrations of FSEO. Basically, 3 mL of Tween 80 was mixed with 97 mL of sterile distilled water. FSEO at 25, 50, 100, 200 and 400 μL/mL was prepared by adding 25, 50, 100, 200 and 400 mL of FSEO each to 1 L of sterile distilled water and Tween 80 (3%), respectively [31]. The culture plates were incubated at 25 °C for 7 days, and the zones of inhibition were observed and measured. All experiments were performed in triplicate.

#### 2.4.2. Radial Growth Method

Radial growth of *F. solani* was evaluated at different concentrations of FSEO (25, 50, 100, 200 and 400 μL/mL) according to method used by Hashem et al. [1], with minor changes. The fennel essential oil was mixed well with the molten CZA medium at the desired final concentrations. Different concentrations of essential oil were prepared by dissolving the required amounts in sterile CZA amended with Tween 80 (0.1%, *v*/*v*) to obtain the desired concentrations (25, 50, 100, 200, 400 µL/mL). The medium was then poured into Petri dishes and kept until solidifying. The center of each plate was inoculated with a mycelium plug (6 mm diam.) from a 7-day-old culture, and the plates were then incubated at 25 ± 2 °C. Mycelium growth was assessed daily by measuring the diameters of the colony in each plate. Inhibition percentage of pathogen growth was calculated using the following equation:

$$\mathrm{Inhibition}\;\mathrm{of}\;\mathrm{pathogen}\;\mathrm{growth}\;\left(\%\right)=\frac{\mathrm{Growth}\;\mathrm{in}\;\mathrm{the}\;\mathrm{control}-\mathrm{Growth}\;\mathrm{in}\;\mathrm{the}\;\mathrm{treatment}}{\mathrm{Growth}\;\mathrm{in}\;\mathrm{the}\;\mathrm{control}}\times 100$$

### 2.5. Pot Experiment

#### 2.5.1. Preparation of Fungal Inoculum 

The inoculum of *F. solani* KHA10 was prepared based on Büttner et al. [32] with slight modification as follows: a 500 mL sterilized Erlenmeyer flask containing 250 mL of the sterilized CZ medium was inoculated with 3 discs (5 mm in diameter) from the edge of 5-day-old *Fusarium* culture, and then incubated in the dark for 7 days at 25 ± 2 °C under shaking (125 rpm). Conidiospores were counted using a hemocytometer, and the inoculum suspension was adjusted to a final concentration of 10^6^ spores/mL. The inoculum was kept chilled at 4 °C until use.

#### 2.5.2. Fennel Seeds, Growth Conditions and Treatments 

Seeds of *Vicia faba* L. (Nubaria1) were obtained from the Agriculture Research Center (ARC), Ministry of Agriculture, Egypt. The *Vicia faba* seeds were washed with distilled water then sterilized using 2% sodium hypochlorite for 2 min. *Vicia faba* seeds were grown in plastic pots (15 cm in diameter × 15 cm in depth), previously sterilized using a 5% formaldehyde solution and filled with 1 kg of sterile sandy clay soil (4:1). Two weeks before planting, the soil was infested with *F. solani* KHA10. Soil infestation was carried out by adding 90 mL of a 10^6^ spore/mL suspension of *F. solani* KHA10/pot. The infested soil was kept moist for 7 days to stimulate fungal growth and ensure homogeneous distribution of the fungus. The control treatment was prepared by adding the same amount of the sterilized Czapek-Dox broth (CZ) to the sterilized soil of each pot. The pots were grown in greenhouse conditions at 25 ± 5 °C, with a 14 ± 2 h light regimen and humidity at 65 ± 10%, and irrigated as necessary. Treatments used in this study were as follows: (1) healthy control (C)—the sterilized *Vicia faba* seeds submerged in distilled water (D.W.) for 6 h and sowing in sterilized soil; (2) treated with FSEO (T)—the sterilized *Vicia faba* seeds soaked in FSEO 400 µL/mL at the minimal fungal concentration (MFC) for 6 h and sown in sterilized soil; (3) control infected with *F. solani* (P)—sterilized *Vicia faba* seeds soaked in D.W. for 6 h and sown in soil previously inoculated with *F. solani*; (4) seeds treated with FSEO (T+P), sterilized *Vicia faba* seeds soaked in FSEO 400 µL/mL for 6 h and sown in soil previously inoculated with *F. solani*. The data were collected at 3 intervals (3 weeks, 6 weeks and 10 weeks). The results collected after 3 weeks of growth were much like the control because there was not much time for plant to be affected by both the pathogen and the treatments. After 10 weeks, it was very hard to collect data because the plant was old and affected too much by the pathogen to create a huge variation in the results (Supplementary Figure S1, Tables S1–S3). Five seeds/pot of *Vicia faba* for each treatment were applied. All experiments were arranged in a completely randomized split-plot design with 3 replicates per treatment. Six weeks after planting, all pots were evaluated for the incidence of *Fusarium* root rot. Percentages of seed rot, pre- and post-emergence damping off, and plant survival were also recorded [33].

### 2.6. Disease Assessments

Disease severity (DS) and incidence (DI) of *Fusarium* root rot were assessed in *Vicia faba* L. 6 weeks after planting. Disease severity was evaluated using the 0–5 scale described by [34].

Disease severity (%) = ∑ab/AK × 100

where a = number of diseased plants with the same infection degree, b = infection degree, A = total number of the evaluated plants and K = the greatest infection degree. 

Disease incidence (DI) of *Fusarium* root rot was assessed pre-emergence and at post-emergence damping off after the treatments. Disease incidence was calculated for each treatment according to the following equation:

Disease incidence (%) = a/A × 100

where, a = number of diseased plants and A = total number of evaluated plants.

### 2.7. Analysis of Plant Growth Parameters

Samples were assessed after 6 weeks of sowing. The morphological traits of treated and untreated faba bean plants were measured. Three plants with *Fusarium* root rot from each experiment were harvested and transferred to the laboratory, carefully uprooted and washed using tap water for measuring plant height, and shoot and root fresh and dry weight. For dry weight, samples were oven-dried at 40 °C for 48 h. 

### 2.8. Biochemical Analyses

For each treatment, 3 plants were collected 30 days after treatment and analyzed for total phenol content (TPC), total flavonoid content (TFC), phenylalanine ammonia lyase (PAL), polyphenol oxidase (PPO), 2,2-diphenyl-1-picrylhydrazyl (DPPH) and antioxidant enzymes.

#### 2.8.1. Determination of 2,2-diphenyl-1-picrylhydrazyl (DPPH) Radical Scavenging Activity

The scavenging activity of DPPH radicals was evaluated by adding a 1 mM solution of DPPH in ethanol to 1.5 mL (1 mg/L mL) of the EO extract solution. The freshly prepared DPPH solution was taken in test tubes and extracts were added, followed by serial dilutions (100–1000 μg) in every test tube such that the final volume was 2 mL, and the absorbance was evaluated at 517 nm against the corresponding blank solution, which was prepared by taking 3 mL ethanol, and the control O.D. was prepared by taking 3 mL of DPPH. The assay was repeated 3 times. DPPH percentage inhibition was estimated based on the control reading [1].

DPPH scavenged (%) = (A cont. − A test)/A cont. × 100

where A cont. is the absorbance of the control reaction and A test is the absorbance in the presence of the sample of the extracts.

#### 2.8.2. Total Phenolic Content

Total phenols were measured in the uppermost leaves using the ethanol extraction method (80%, *v*/*v*); the supernatant was added to Folin and Ciocalteau’s reagent as described [35]. 

#### 2.8.3. Total Flavonoid Content

Total flavonoid content (mg·g^−1^ fresh weight) was measured using aluminum chloride catechin equivalent (CAE) as the standard accordingly [35]. 

#### 2.8.4. Phenylalanine Lyase Assay

PAL activity was determined following the method described by Whetten and Sederoff [36]. The mixture of the assay, including 500 μL 50 mM Tris HCI and 100 μL plant extract, (pH 8.8), and 600 μL 1 mM L-phenylalanine, was incubated at room temperature for 1 h, and 2 N HCI was used to stop the reaction. Toluene (1.5 mL) was used to extract the assay mix by vertexing for 30 s. After centrifugation at 300 g, toluene was recovered for 5 min using a CRU-5000 centrifuge ITC. The toluene phase (containing trans-cinnamic acid) absorbance was measured at 290 nm. The enzyme activity was expressed as nmol trans-cinnamic acid released min^−1^ g^−1^ fresh weight.

#### 2.8.5. Polyphenol Oxidase (PPO)

Extraction of PPO was performed as reported by [37]. Powdered samples (0.5 g) were homogenized with a buffer containing 20 mL of a 100 mM sodium phosphate buffer (pH 7.0) and 0.5 g polyvinyl pyrrolidone (PVP) (mol. wt 40,000) for the assay of the activity of PPO. The activity was measured in powder extracted with a 50 mM sodium phosphate buffer (pH 8.8) containing 5 mM β-mercaptoethanol. The extracts were filtered through 2 layers of Mira cloth, and the filtrates were centrifuged at 27,000× *g* at 4 °C for 30 min.

#### 2.8.6. Antioxidant Enzyme Quantification 

Samples (500 mg) of leaves were homogenized in a 50 mM KH_2_PO_4_ buffer (pH 7.8) with 0.1 mmol L^−1^ EDTA, 0.1% (*v*/*v*) Triton X-100 and 2% PVP, and centrifuged at 4 °C for 10 min at 22,000× *g*. The supernatant obtained was reserved for the assays of the different antioxidants. 

The activities of total superoxide dismutase (SOD, EC 1.15.1.1), catalase (CAT, EC 1.11.1.6) and ascorbate peroxidase (APX, EC 1.11.1.11) were recorded as follows: SOD activity was evaluated based on Kono (1978) [38] by measuring its ability to inhibit the photochemical reduction of nitroblue tetrazolium (NBT). The reduction of NBT was followed by an absorbance increase at 540 nm in a reaction mixture containing 1.3 mL Na-carbonate buffer (50 mM, pH 10.0), 500 µL NBT (96 µM) and 100 µL Triton X-100 (0.6%). The reaction was initiated by the addition of 100 µL hydroxylamine-HCl (20 mM, pH 6.0); 2 min later, 70 µL of the enzyme sample was added. The enzyme activity was calculated as the SOD concentration inhibiting the reduction of NBT by 50%. CAT activity was measured based on the method described by Aebi (1974) [39]. The rate of decomposition of H_2_O_2_ was superseded by a decrease in absorbance at 240 nm in a reaction mixture containing 1.5 mL K-phosphate buffer (100 mM, pH 7.0), 1.2 mL H_2_O_2_ (150 mM) and 300 µL of the enzyme extract. Enzyme activity was estimated by the extinction coefficient of 6.93 × 10^−3^ mM^−1^ cm^−1^. Moreover, APX activity was measured based on the method of Nakano and Asada (1981) [40] achieved by a decrease in absorbance at 290 nm in a reaction mixture containing 1.5 mL K-phosphate buffer (100 mM, pH 7.0), 300 µL ascorbate (5 mM), 600 µL H_2_O_2_ (0.5 mM) and 600 µL of the enzyme extract. Enzyme activity was determined using the extinction coefficient of 2.8 mM^−1^ cm^−1^, and was calculated as the amount of enzyme required to oxidize 1 µmol of ascorbate min^−1^ g^−1^ tissue.

#### 2.8.7. Expression of Defense-Related Genes

Total RNA was extracted from 0.5 g fresh faba leaves at 1, 2 and 3 weeks after sowing from all treatments and the control using an RNA extraction kit (QIAGEN, Redwood, CA, USA). The obtained RNA was incubated with DNase for 1 h at 37 °C and quantified using a NanoDrop 1000 spectrophotometer (Thermo Scientific, Waltham, MA, USA). An RT-PCR kit (Omniscript RT; QIAGEN) was used for the synthesis of cDNA. Thermo QuantStudio 12K Flex Real-Time PCR System qRT-PCR was carried out in triplicate with 3 biological repeats using TOP real TM qPCR 2X Pre MIX SYBR Green (Enzynomics, Daejeon, Korea) according to the manufacturer’s instructions using the given primers of the defense-related genes defensin and chitinase (Table 1) using β-actine as the reference gene. The PCR cycle was: 95 °C for 5 min (hot-start activation) followed by 40 cycles of 95 °C for 10 s (denaturation), 58 °C for 20 s (annealing) and 72 °C for 20 s (extension). The melting curve was generated after 40 cycles to test the specificity of each primer pair across the temperature range of 60–95 °C at a heating rate of 0.05 °C/s. Gene expression analyses were performed according to Rawat et al. [41].

### 2.9. Statistical Analysis

Data Procession System (DPS) was used for analysis of variance (ANOVA). Two-way ANOVA was used to test the effect of E and P and their interactions on plant health, followed by the least significant difference (LSD). Correlation, PCA analysis and presentation were performed using R version 3.4.2. 

## 3. Results

### 3.1. Chemical Composition of Fennel Oil

**Figure 1:**
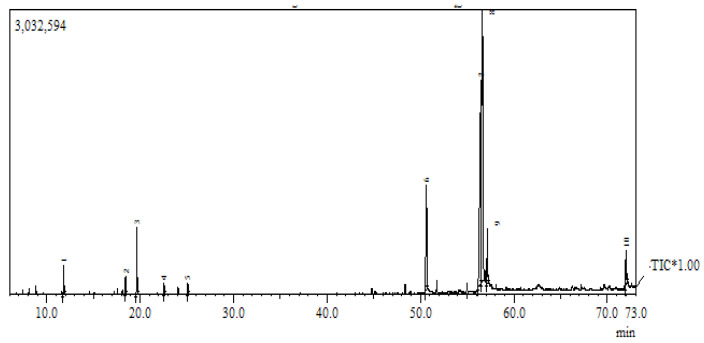
Chromatogram: GC-MS chromatogram of *Foeniculum vulgare* essential oils.

Since the researchers did not know the mechanism behind the antifungal activities of fennel seeds, this study attempted another experiment to check the chemical composition of fennel seeds and whether it could lead us to a significant result. Therefore, the chemical composition of fennel oil was inspected through gas chromatography/mass spectrometry (GC/MS) analysis, which revealed the presence of 10 major compounds in different percentages. The most abundant compound was cis-vaccenic acid (31.23%), followed by 9,12 octadecadienoic acid (29%), pentadecanoic acid (7.51%), estragole (4.39%), octadecadienoic acid (3.92%); 9-octadecadienoic acid (3.75%), D-limonene (2.93%), menthol (1.89%), 2,4-decadienal (1.68%) and 2-decenal (1.58%) (Figure 1 and Table 2).

### 3.2. In Vitro Control of F. Solani by Fennel Essential Oils

#### 3.2.1. Antifungal Activity and Minimum Inhibitory Concentration of FSEO

**Figure 2:**
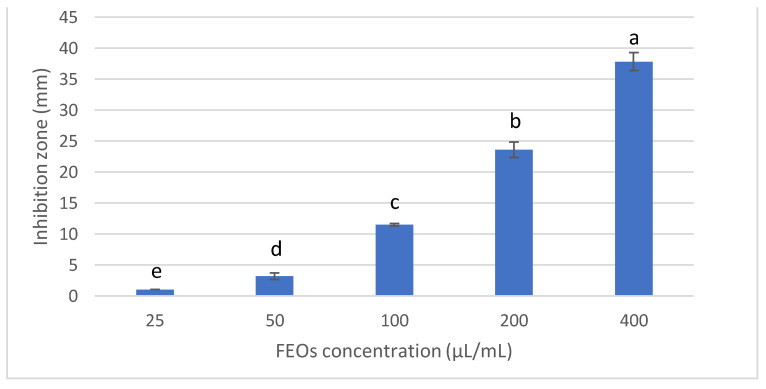
Antifungal activity of FSEO at different concentrations against *F. solani*. Data are expressed as means ± standard deviations in triplicate. Different alphabetic superscripts in the same column are significantly different (*p* < 0.05) based on Tukey’s multiple comparison test.

The antifungal activity of fennel seeds was investigated at different concentrations (25, 50, 100, 200 and 400 µL/mL) to inhibit *Fusarium solani* KHA10, the causative agent of *Fusarium* root rot disease in *Vicia faba* by the agar well diffusion method (Figure 2). The results revealed that all concentrations of FSEO showed antifungal activity against *F. solani* KHA10. However, 400 µL/mL presented the most antifungal activity, with a 38 mm inhibition zone, while 25 µL/mL exhibited the lowest antifungal activity, inhibiting the growth of *F. solani* with a 1 mm inhibition zone. According to the previous results, 25 µL/mL of FSEO was the MIC for controlling *F. solani*.

#### 3.2.2. Effect of FSEO on the Radial Growth of *F. solani* and Minimum Fungicidal Concentrations

**Figure 3:**
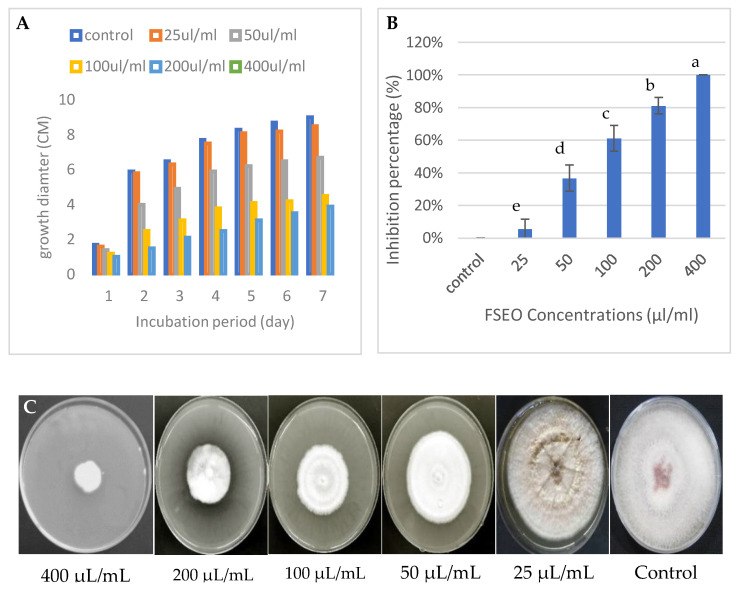
Effect of FSEO on *F. solani* (**A**–**C**): (**A**) radial growth at different incubation periods from 1 to 7 days; (**B**) inhibition percentages of *F. solani* at different concentrations of FSEO; (**C**) radial growth on Czapek-Dox agar medium (CZA) plates at 7 days. CZA not supplemented with FSEO was used as a control. Data are expressed as means ± standard deviations of triplicate assays. Different alphabetic superscripts in the same column are significantly different (*p* < 0.05) based on Tukey’s multiple comparison test.

In vitro, the antifungal activity of different concentrations of fennel seed essential oil extract was tested against the mycelial growth of *F. solani* KHA10 with different incubation periods from 1 to 7 days (Figure 3A–C). The radial growth was examined to measure the inhibition percentage of each FSEO concentration. The results demonstrated that the inhibition percentage increased with an increasing concentration of FSEO, while the radial growth ceased, as shown in Figure 3B. *Fusarium solani* could not grow at 400 µL/mL on the CZA surface, with an inhibition percentage of 100%, so this concentration had the minimum fungicidal activity (Figure 3). Moreover, FSEO at 25 µL/mL allowed the growth of *F. solani* with only 6% inhibition percentage. 

### 3.3. In Vivo Control of F. solani KHA10 by Fennel Essential Oil

#### Efficacy of FSEO on *Fusarium* Root Rot Disease of *Vicia faba* L. under Pot Conditions

After applying the treatments to *Vicia faba*, morphological and disease progression were observed at the pre-emergence, post-emergence and 6 week stages (Table 2). Pre- and post-emergence, the damping off was decreased by 25% and 19%, respectively. The seeds exposed to the pathogen and FSEO (P+E) were much healthier compared were seeds treated with the pathogen (P) only. In addition, the P-only treated seeds clearly developed the disease. 

The growth performance of FSEO-treated plants were much improved over the control (C) and P plants after 6 weeks of plantation. Broad bean plants treated with P+E were taller and healthier than P plants. The results showed that the disease resistance was higher in the 400 µL/mL FSEO treated plants. 

After 400 µL/mL FSEO treatment of *Vicia faba* L. seeds, disease incidence (DI) and disease severity (DS) decreased significantly as compared with the pathogen-inoculated seeds only at 6 weeks after planting. The percentage of DI in faba seeds soaked with 400 µL/mL FSEO decreased to 33.5% as compared with pathogen-only infected plants. Plant survival and protection were clearly improved when FSEO was applied to infected seeds by approximately 44% and 50%, respectively (Table 3). 

### 3.4. Physiological Characterization of FSEO Treated Faba Bean Plants

The physiological characterization data of greenhouse application treatments showed a significant increase in the growth parameters of *Vicia faba* plants, viz. plant height (P.h), shoot fresh weight (SFW), root fresh weight (RFW), shoot dry weight (SDW) and root dry weight (RWD), by soaking seeds in 400 µL/mL FSEO, compared with *F. solani* KHA10 inoculation. Maximum P.h was recorded in the case of FSEO: 49.92 cm at 6 weeks after sowing. The control was second in rank, where a P.h of 43.20 cm was recorded, while T+P recorded a P.h. of 39.50 cm. As expected, the lowest P.h was observed in the case of the pathogen treatment: 36.70 cm at 6 weeks after sowing. Overall, SFW, RFW, SDW and RDW were significantly higher in the T+P treatment than in the pathogen-only treatment. Apart from that, we also observed a significant in of SFW, RFW, SDW, and RDW in the oil-treated plants as compared with the controls (Table 4).

### 3.5. Influence of FSEO on Different Biochemical Parameters

**Figure 4:**
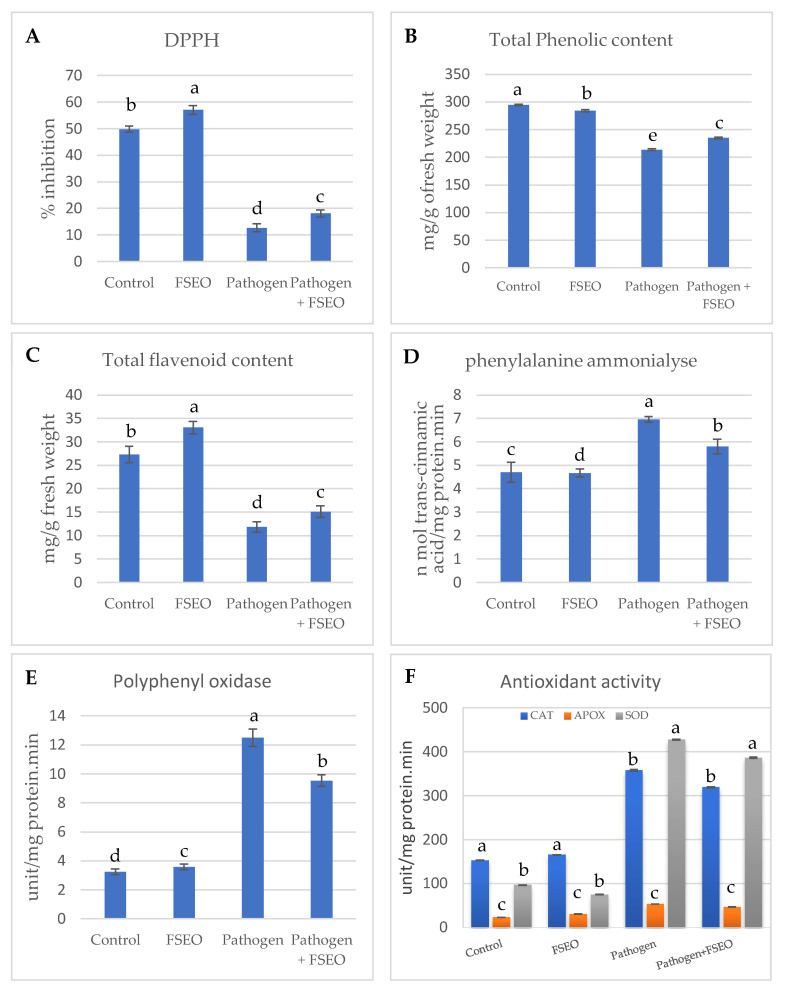
Biochemical components and antioxidant enzymes of plants, after 6 weeks of treatment. 2,2-Diphenyl-1-picrylhydrazyl (DPPH) radical scavenging activity (**A**), total phenol content (TPC) (**B**), total flavonoid content (TFC) (**C**), phenylalanine ammonia lyase (PAL) (**D**), polyphenol oxidase (PPO) (**E**) and antioxidant enzymes (**F**). Error bars indicate ± standard error of the mean of three replicates. Different alphabetic superscripts in the same column are significantly different (*p* < 0.05) based on Tukey’s multiple comparison test.

The second part of this study was the biochemical analysis of fennel seed extract and its influence on 2,2-diphenyl-1-picrylhydrazyl (DPPH), total phenolic contents (TPC), total flavonoid contents (TFC), phenylalanine ammonia lyase (PAL) and polyphenyl oxidase (PPO) (Figure 4). Significantly high DPPH was detected for *Vicia faba* L. treated with FSEO, which measured 57.05 μg g^−1^ dry wt. 6 weeks after sowing, while the control recorded 49.97 μg·g^−1^ dry wt. (Figure 4A). Pathogen-infected *Vicia faba* L. was strongly affected, where DPPH was 12.77 μg g^−1^ dry wt., illustrating the strong influence of the pathogen on faba bean plants. A significant decrease in DPPH values was reported in the case of FSEO+P, namely 18.16 μg g^−1^ dry wt., explaining the role of FSEO in plants defense against fungal diseases (Figure 4A).

Total phenolic content is another biochemical contributor in plants that has redox properties, acting as an antioxidant (Figure 4B). The highest TPC was detected in control plants 6 weeks after sowing, recording 294.68 mg catechol 100 g^−1^ dry wt., while in the case of the FSEO treatment, the recorded TPC was 284.34 mg catechol 100 g^−1^ dry weight. In the case of FSEO+P, the recorded TPC was 235.13 mg catechol 100 g^−1^ dry wt. Total phenolic content dramatically decreased to 213.83 mg catechol 100 g^−1^ dry wt. when plants were treated with *F. solani* (pathogen) (Figure 4B). 

The level of total flavonoid content increased in *Vicia faba* L. treated with FSEO to 33.06 mg rutin 100 g^−1^ dry wt. at 6 weeks after sowing, while the control was 27.29 mg rutin 100 g^−1^ dry wt. The treatment of faba beans with FSEO +P recorded TFC at 15.09 mg rutin 100 g^−1^ dry wt., while the level of TFC was significantly decreased at 11.84 mg rutin 100 g^−1^ dry wt. in the case of the pathogen treatment (Figure 4C).

Additionally, the highest assay of PAL was detected when *Vicia faba* L. was treated with pathogen: 7.00 nM cinnamic g^−1^ fresh wt. 6 weeks after sowing, while PAL was at 4.7 nM cinnamic g^−1^ fresh wt. in the case of the control. PAL was at 5.81 nM cinnamic g^−1^ fresh wt. when faba bean was treated with FSEO +P, whereas PAL was at 4.67 nM cinnamic g^−1^ fresh wt. in the case of FSEO (Figure 4D).

Regarding polyphenol oxidase (PPO), the data showed a significantly increase PPO 12.50 μg g^−1^ dry wt. when *Vicia faba* L. was treated with *F. solani*. Moreover, PPO was at 9.55 μg g^−1^ dry wt. in case of FSEO +P, while FSEO only recorded 3.57 μg g^−1^ dry wt. The smallest amount of PPO, 3.24 μg g^−1^ dry wt., was recorded with the control (Figure 4E).

**Figure 5:**
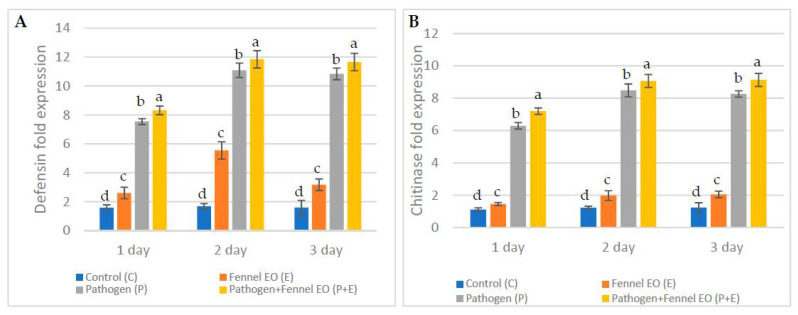
Fold expression in the accumulation of the defense-related genes (**A**) defensin and (**B**) chitinase in *Vicia faba* L. samples, at different treatments relative to the control, and at different periods. Values are the means (±SD) of three repeated experiments. Different alphabetic superscripts in the same column are significantly different (*p* < 0.05) based on Tukey’s multiple comparison test.

In addition, DI showed a strong negative (*p* ≤ 0.01) correlation with biochemicals such as DPPH, TPC and TFC, illustrating that an increase in disease incidence or severity will lead to a decrease in these biochemical or plant physiological characteristics and vice versa. However, there was a strong positive (*p* ≤ 0.01) correlation between DI with antioxidants, showing that a parallel increase or decrease in one will affect the other component positively (Figure 5).

**Figure 6:**
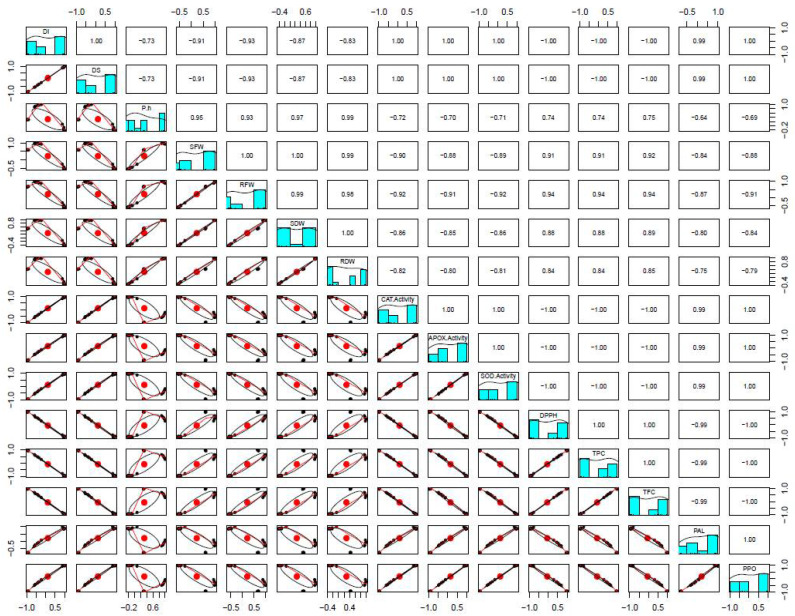
Correlation coefficients (*r*) between different plant parameters. The *r* values from 0.50–0.70 have *p* ≤ 0.05 and those from 0.70–1.0 have *p* ≤ 0.01.

Concurrently, we also performed PCA analysis to identify the relationship of variables at 6 weeks in plants grown under different treatments. Correlations between variables were found via biplot analyses, where an acute angle means a positive correlation, an obtuse angle means a negative correlation and a right angle means no correlation between the measured parameters. The first principal component has the largest variance due to the orthogonal transformation. According to the PCA calculated for all the data, the first factor (PC1) explained 71.5% of the total variance of the variables, and the second factor (PC2) about 24.2%. In total, both PCs explained 95.7% of the total variance of all the analyzed variables (Figure 6).

### 3.6. Expression Levels of Defense-Related Genes

Pathogenesis-related genes are of great importance in plants that have greatly raised the level of their defense mechanisms against a wide range of pathogens. Therefore, in the third part of the current study, defensin and chitinase gene expression (GE) levels were evaluated. Not surprisingly, both genes showed highly significant levels of expression in the FSEO +P and P treatments, illustrating activation of the defense-related machinery in these treatments. In particular, defensin GE was significantly high (11.84, 11.65, 11.08 and 10.83) for the FSEO +P and P treatments on the second and third day, respectively. On the other hand, significantly low defensin GE levels were seen for other treatments. Accordingly, chitinase GE levels were significantly high (9.13, 9.07, 8.48, 8.26 and 6.29) in the FSEO +P and P treatments on the second and third day, while the other treatments showed relatively low chitinase GE levels (Figure 5).

## 4. Discussion 

The resistance of many crops to fungicides continues to cause serious disease control problems. The practical research experience gathered over the past 50 years has highlighted the importance of using different strategies to control plant diseases. Moreover, scientific research is becoming alarmed not only due to losses from pathogen resistance but also environmental and health concerns. Therefore, there has been increasing interest in the serious pursuit of alternative biological phyto-therapeutic agents. Thus, this study is an attempt toward evaluating the applicability and the potential of essential oils derived from *Foeniculum vulgare* Mill. in the control of *Fusarium* root rot disease in *Vicia faba* L. under greenhouse conditions.

In the present study, we reported the antifungal activity of FSEO against *Fusarium solani* KHA10 in vitro and in vivo. Through GC-MS analysis of oil from *Foeniculum vulgare*, 10 different components were identified. In another study, gas chromatography of essential oils showed the presence of 18 main monoterpenoids in fennel oil. Limonene, trans-anethole, fenchone and estragole were common in fennel oil [21]. Another study reported that the volatile oil of fennel contains different components: fenchone (1–20%), anethole (40–70%) and estragole (2–9%) [33]. Many other GC-MS screenings enumerated different components: fenchone (1–20%), estragole (2–9%) and anethole (40–70%) [42]. Similarly, in another experiment, fennel essential oil contained fenchone (less than 5%), but bitter types contained 20%. Sweet fennel oil contains 84–90% anethole, but bitter fennel contains 61–70% [18]. In this respect, the fennel seed essential oil, which contained a high amount of menthol, showed good antifungal activity against *F. solani*, as shown in previous results [33]. 

The results of the current study showed that fennel seed essential oils suppressed mycelium growth of *Fusarium solani* KHA10 in vitro at different concentrations from 25 to 400 µL/mL. Evidently, the inhibition of fungal growth increased with the increase in the concentration of the essential oil [33]. Plant-derived essential oils are compounds that have antibacterial and antifungal activity [15]. An illustration of this is a research study on *Botrytis cinerea*, in which different concentrations of essential oils promisingly and effectively suppressed the growth of *Botrytis cinerea* in a dose-dependent manner [26]. Our revealed data are in high accordance with the findings on another organism [43], which stated that the disease occurrence of powdery mildew on *Zinnia elegans* was significantly diminished through spraying with ginger, cinnamon, fennel and clove essential oils.

Another compelling piece of evidence was found from a research study investigating the high potential antifungal ability of essential oils of *Artemisia indica*, *Mentha spicata*, *Eucalyptus citriodora*, *Cymbopogon citratus* and *Cinnamomum tamala*, recording highly significant activity against *Fusarium oxysporum* and *Aspergillus niger* [44]. Among the tested essential oils, *Cymbopogon citratus* displayed the highest productive antifungal potential against *Fusarium oxysporum* (100% inhibition in mycelial growth) at 40 μL mL^−1^. *Mentha spicata* showed potent antifungal activity against *Aspergillus niger* (92.93% inhibition in mycelial growth) at 40 μL mL^−1^ concentration [44]. The possible mechanism of action could be attributed to the disruption of the plasma membrane and disorganization of the mitochondrial structure caused by essential oils, as previously reported [45]. Therefore, a recent confirmative hypothesis reported that essential oils contain specific antifungal compounds and fungitoxic agents that inhibit the growth of certain microorganisms [45,46].

The current study reported a significant change in the physiological and morphological characteristics of *Vicia faba* L. plants treated with *F. solani* KHA10, confirming previously reported findings [30], which also stated that the morphological characteristics of asparagus were significantly reduced after inoculation with *Fusarium* species. In this study, the efficacy of fennel seed essential oil was examined in *Vicia faba* L. under greenhouse conditions. Both curative and preventive oil treatments were effective in reducing *F. solani* KHA10 infection. The disease incidence and severity were obviously decreased when plants were treated with FSEO.

Moreover, plant polyphenols are major compounds produced by plants for resistance to pathogens and many other functions [47]. In the present study, total phenolic and flavonoid contents were significantly increased when FSEO was applied on inoculated and non-inoculated faba bean plants when compared with seeds not treated with FSEO and sown in infested soil (pathogen). Similar results were described by [15,34,47], who stated that the phenolic compounds may prevent infection by the pathogen by increasing the mechanical strength of the host’s cell walls and thus inhibiting pathogen infection.

Additionally, plant essential oils have unique antioxidant and antimicrobial properties, and are recommended to be a good alternative to synthetic antioxidants and chemical pesticides [48,49]. In this research, inhibition of the fungal activity using essential oil of fennel led to a significant increase in the fresh and dry weight of the shoot and root system of *Vicia faba* L. plants. There was a clear significantly negative relationship (Figure 6) between the degree of disease severity and fresh plant weight, indicating that infection with *F. solani* KHA10 was the main growth-limiting factor in all plants in the present and previous studies [1,25].

In order to evaluate the molecular mechanisms concerned in FSEO-induced resistance in faba bean, the expression of two defense-related genes, defensin and chitinase, was assessed in treated faba bean at different times. It is worth mentioning here that the expression of defense-related genes can be induced by pathogen inoculation and environmental stresses [33]. The identification of such a broad mechanism involved in defense against pathogens and environmental stresses provides new opportunities for crop improvement. Plant defensins are a family of cysteine-rich peptides, many members of which have been shown to exhibit antimicrobial activity against various microbial attacks [50]. In the present study, we reported the high expression of defensin and chitinase GE treated with the pathogen and FSEO after the third day. These finding show that FSEO may also act as an inducer of the defense-related genes of plants when co-applied with pathogens.

Supporting such findings, it is worth mentioning that essential oils have two advantages: they are safe for use and have a low risk of the microorganisms developing resistance [46]. Essential oils are biodegradable and non-toxic. Since these bioactive compounds are extracted from plants, they are thought to be more acceptable and less risky to the environment than synthetic compounds [10]. In fact, different essential oils as antioxidants are naturally found in plants and have been considered as scavengers of active oxygen [13]. Due to the hydroxyl groups, phenolic compounds play an essential role in their scavenging ability [34,35,50]. Several reports highlighted the rapid advancement of essential oils as biodegradable, less toxic and eco-safe fungi-toxicants, showing the possibilities for their exploitation as natural fungicides [21]. 

## 5. Conclusions

Fennel seed essential oil, applied at a concentration of 400 μL/mL, has antifungal activities against *Fusarium solani* KHA10 in vitro and in vivo. The defensin and chitinase gene profile indicated that these genes may play vital roles in the resistance mechanism via reducing *Fusarium* root rot in faba beans. Although more studies are needed to fully verify and understand its mode of action, fennel seed oil is a promising fungicide against *F. solani* KHA10 as well as a plant growth promoter.

## Figures and Tables

**Table 1:** Primers used for qRT-PCR defense gene analysis.

Primer	Primer Sequence	Annealing Temp.
Chitinase-F	5′-GCGGATCCCAACGCACTGCAACCGATTAT-3′	60 °C
Chitinase-R	5′-GCCCATGGAAGGAATCAGTTATGCGCAAAT-3′	
Defensin-F	5′-CCAAATGCCTCGTCATCT-3′	
Defensin-R	5′-ATTAGAGTCAAGCTCAAAAGG-3′	
β-Actin	5′-GTGGGCCGCTCTAGGCACCAA-3′	
β-Actin	5′-CTCTTTGATGTCACGCACGATTTC-3′	

**Table 2:** Chemical composition of *Foeniculum vulgare* oil by GC-MS analysis.

Quantitative ID	Component Identified	Retention Time (min)	Area (%)
1	D-limonene	11.81	2.93
2	Menthol	18.44	1.89
3	Estragole	19.66	4.39
4	2-Decenal	22.51	1.58
5	2,4-Decadienal	25.1	1.68
6	Pentadecanoic acid	50.64	7.51
7	9,12-Octadecadienoic acid	56.45	29.49
8	Cis-vaccenic acid	56.63	31.23
9	Octadecadienoic acid	57.15	3.92
10	9-Octadecadienoic acid	72	3.75

**Table 3:** Mean *Fusarium* root rot incidence and severity at pre- and post-emergence damping off after different treatments were applied to *Vicia faba* L.

Treatments	Pre-Emergence Damping off %	Post-Emergence Damping off %	Survival Plant %	Disease Severity (DS) (%)	Disease Incidence (DI) %	Protection %
Healthy control (C)	0	0	100	0	0	-
Treated with FSEO (T)	0	0	100	0	0	-
Infected control (P)	46.5	33.8	19.71	53.1	67.4	0
Treated +infected (T+P)	21.8	14.5	63.7	20.6	33.5	32.5

**Table 4:** Effect of FSEO and *F. solani* KHA10 on morphological parameters of *Vicia faba* L. in pot conditions at 6 weeks of treatment.

Treatments	Plant Height (cm)	Shoot F. wt. (g)	Root F. wt. (g)	Shoot D. wt. (g)	Root D. wt. (g)
Healthy control (C)	43.2 ± 0.18 ^d^	4.37 ± 0.02 ^b^	2.16 ± 0.03 ^a^	0.56 ± 0.03 ^a^	0.34 ± 0.07 ^a^
Treated with FSEO (T)	49.92 ± 1.2 ^a^	4.75 ± 0.03 ^a^	2. 43 ± 0.03 ^a^	0.66 ± 0.02 ^b^	0.38 ± 0.06 ^b^
Infected control (P)	36.7 ± 1.0 ^b^	2.69 ± 0.02 ^b^	1.59 ± 0.02 ^b^	0.38 ± 0.01 ^c^	0.24 ± 0.02 ^c^
Treated + infected (T+P)	39.4 ± 0.52 ^c^	3.27 ± 0.02 ^b^	1.74 ± 0.03 ^a^	0.41 ± 0.01 ^d^	0.28 ± 0.01 ^d^

Values are the means of 15 replicates ± standard errors. Values in each column followed by the same letter are not significantly different according to Duncan’s multiple range test (*p* ≤ 0.05).

## Data Availability

The study did not report any data.

## Supplementary Materials

The following are available online at https://www.mdpi.com/article/10.3390/biology10080696/s1, Figure S1: In vitro inhibitory effect of 400 μL/mL of FSEO against *F. solani*, Table S1: Mean Fusarium root rot incidence and severity in pre- and post-emergence damping off after different treatments were applied to *Vicia faba* L. after 3 weeks of treatments, Table S2: Effect of FSEO and F. solani KHA10 on morphological parameters of *Vicia faba* L. under pot conditions at 3 weeks of treatments, Table S3: Biochemical components and antioxidant enzymes of plants, after 6 weeks of treatment.
