# Supplementary material for: Essential Oil of Foeniculum vulgare Mill. as a Green Fungicide and Defense-Inducing Agent against Fusarium Root Rot Disease in Vicia faba L
Source: Biology (Basel). 2021 Jul 22;10(8):696. doi: 10.3390/biology10080696 (PMC8389234; doi:10.3390/biology10080696)
Supplement: Supplementary file 1 [file biology-10-00696-s001.zip › biology-1294590-supplementary.pdf]

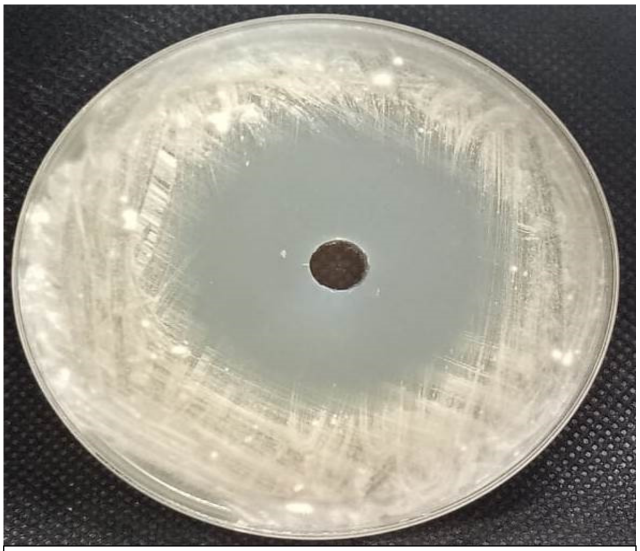

Figure S1. *In vitro* inhibitory effect of 400µl/ml of FSEO against *F. solani*

Table S1. Mean *Fusarium* root rot incidence and severity in pre- and post-emergence damping off after different treatments were applied to *Vicia faba* L. after 3 weeks of treatments.

Treatments	Pre-Emergence Damping off %	Post-Emergence Damping off %	Survival Plant %	Disease severity (DS) (%)	Disease incidence (DI) %	Protection %
Healthy control (C)	0	0	100	0	0	-
Treated with FSEO (T)	0	0	100	0	0	-
Infected control (P)	21.4	14.6	67.72	10.1	29.4	21.1
Treated +infected (T+P)	17.7	11.2	53.7	20.6	31.5	18.5

Table S2. Effect of FSEO and *F. solani* KHA10 on morphological parameters of *Vicia faba* L. under pot conditions at 3 weeks of treatments

Treatments	Plant Height (cm)	Shoot F. wt. (g)	Root F. wt. (g)	Shoot D. wt. (g)	Root D. wt. (g)
**Healthy control (C)**	33.2	3.1	1.16	0.46	0.34
**Treated with FSEO (T)**	34.90	3.0	1.3	0.46	0.35
**Infected control (P)**	32.7	3.17	1.59	0.41	0.29
**Treated +infected (T+P)**	33.4	3.18	1.74	0.41	0.28

Table S3. Biochemical components and antioxidant enzymes of plants, after 6 weeks of treatment. 2,2-diphenyl-1-picrylhydrazyl (DPPH) radicals scavenging activity (A), Total phenol content (TPC) (B), Total flavonoid content (TFC) (C), Phenylalanine ammonialyase (PAL) (D), Polyphenol oxidase (PPO) (E), Catalase enzyme (CAT), ascorbate peroxidase (APX), and Superoxide dismutase (SOD).

Treatments	DPPH	TPC	TFC	PAL	PPO	CAT	APOX	SOD
**Healthy control (C)**	50.08	293.68	27.01	4.51	3.21	151.3	23.14	96.47
**Treated with FSEO (T)**	54.24	290.8	29.07	4.6	3.31	154.86	26.72	98.57
**Infected control (P)**	55.04	283.65	26.4	5.01	3.63	193.1	25.58	111.08
**Treated +infected (T+P)**	52.37	291.4	29.27	4.9	3.73	176.04	23.92	121.82
